# Barriers and Opportunities to Increase Utilization of Donor Kidneys After Death Determined by Circulatory Criteria Among Children and Adults: A Narrative Review

**DOI:** 10.1177/20543581251382333

**Published:** 2025-10-05

**Authors:** Cal Robinson, Adrianna Douvris, Waleed Rahmani, Ayodele Odutayo, Sergi Clotet-Freixas, Ann Young

**Affiliations:** 1 Division of Nephrology, The Hospital for Sick Children, Toronto, ON, Canada; 2 Child Health Evaluative Sciences, Research Institute, The Hospital for Sick Children, Toronto, ON, Canada; 3 Division of Nephrology, Department of Medicine, Ottawa Hospital Research Institute, University of Ottawa, ON, Canada; 4 Division of Nephrology, Department of Medicine, Washington University in St. Louis, MO, USA; 5 Division of Nephrology, University of Toronto, ON, Canada; 6 Division of Nephrology, Sunnybrook Health Sciences Centre, Toronto, ON, Canada; 7 Division of Nephrology, McMaster University and St. Joseph’s Healthcare, Hamilton, ON, Canada; 8 Division of Nephrology, St. Michael’s Hospital, Unity Health, Toronto, ON, Canada

**Keywords:** kidney transplant, donation after circulatory death, ischemic-reperfusion injury, ex vivo preservation, time to death, pediatric transplant, sex considerations

## Abstract

**Purpose of Review:**

Kidney transplantation is associated with survival benefit compared to dialysis. Yet, there is an unmet need for access to kidney transplantation within Canada and globally. Donation after death determined by circulatory criteria (DCC) has expanded access to kidney transplantation among the adult and pediatric population. However, there are concerns inherent to DCC kidneys, including warm ischemia time and ischemia-reperfusion injury (IRI). This narrative review aims to summarize relevant literature in this context, discuss potential opportunities to expand the use of DCC kidneys, and highlight knowledge gaps for further study.

**Sources of Information:**

PubMed (Medline), the Canadian Institute for Health Information, and regulatory bodies for organ donation and transplantation.

**Methods:**

A focused review and critical appraisal of existing literature on the mechanisms of kidney IRI, consideration of sex in experimental studies relevant to DCC kidney transplantation, ex vivo perfusion strategies, and pediatric kidney transplant considerations.

**Key Findings:**

DCC kidneys confer a higher risk of delayed graft function (DGF) due to prolonged warm ischemic time. However, long-term graft survival is generally comparable to that of kidneys from donors with death determined by neurologic criteria (DNC). Key barriers to expansion include the paucity of sex-balanced experimental studies on DCC graft preservation and outcomes and existing protocols for DCC donor selection, including thresholds for warm ischemia time. Ex vivo strategies including non-oxygenated and oxygenated hypothermic machine perfusion and normothermic ex vivo kidney perfusion are promising research areas. Improving DCC protocols to reduce kidney IRI has the potential to further expand access to DCC transplantation, including to the pediatric population.

**Limitations:**

This narrative review only included articles written in English. Study quality was not formally assessed. Discussion points were influenced by the author’s areas of expertise.

## Abrégé

**Motif de la revue:**

La transplantation rénale améliore la survie par rapport à la dialyse, mais l’accès à la transplantation rénale demeure insuffisant au Canada et dans le monde entier. Le don après décès par critères circulatoires (DDCC) a élargi l’accès à la transplantation rénale pour les populations adulte et pédiatrique. Or, les reins issus d’un DDCC soulèvent des préoccupations, notamment en ce qui concerne le temps d’ischémie chaude et les lésions d’ischémie-reperfusion (LIR). Cette revue narrative a pour objectif de résumer la documentation pertinente dans ce contexte, de discuter des possibilités d’étendre l’utilisation des reins issus d’un DDCC et de mettre en évidence les lacunes dans les connaissances nécessitant des recherches plus approfondies.

**Sources de l’information:**

PubMed (Medline), l’Institut canadien d’information sur la santé et les instances réglementaires responsables du don et de la transplantation d’organes.

**Méthodologie:**

Une revue ciblée et une évaluation critique de la documentation existante sur les mécanismes des LIR rénales, la prise en compte du sexe dans les études expérimentales liées à la transplantation de reins issus d’un DDCC, les stratégies de perfusion ex vivo et les considérations propres à la transplantation rénale chez l’enfant.

**Principales observations:**

La transplantation de reins issus d’un DDCC présente un risque plus élevé de retard de la reprise de la fonction du greffon en raison du temps prolongé d’ischémie chaude. Mais la survie à long terme du greffon est généralement comparable à celle des reins de donneurs dont le décès est déterminé par des critères neurologiques (DDCN). Les principaux obstacles à l’expansion comprennent la rareté d’études expérimentales équilibrées selon le sexe sur la préservation et les résultats des greffes de reins issus d’un DDCC, ainsi que les protocoles existants pour la sélection des donneurs de reins issus d’un DDCC, notamment les seuils pour la durée de l’ischémie chaude. Les stratégies ex vivo, comme la perfusion mécanique hypothermique non oxygénée et oxygénée, ainsi que la perfusion rénale ex vivo normothermique, sont des domaines de recherche prometteurs. L’amélioration des protocoles de DDCC visant à limiter les LIR rénales pourrait élargir l’accès à la transplantation, notamment chez les enfants.

**Limites:**

Cette revue narrative n’a inclus que des articles rédigés en anglais. La qualité des études n’a pas été évaluée officiellement. Les points de discussion ont été influencés par les domaines d’expertise des auteurs.

## Introduction

To address long wait times for transplantation, the types of acceptable deceased organ donors have expanded. In addition to organs from donors whose death determination was by neurologic criteria (DNC) (also known as neurological determination of death [NDD] or “brain death”), death determination by circulatory criteria (DCC) has been successfully implemented for organ donation in many parts of the world.^1^ Controlled DCC specifically refers to the use of kidneys from a donor who experiences cardiac arrest following a planned withdrawal of life-sustaining treatment, often in an intensive care unit. Uncontrolled DCC occurs after unexpected cardiac arrest when the donor cannot be resuscitated. In Canada, deceased donor kidney transplants from DCC to adult recipients began in 2006.^2^

One of the main concerns regarding DCC kidneys is that prolonged warm ischemic time—the time from circulatory arrest after withdrawal of life-sustaining treatment to cold perfusion—can result in more severe ischemia-reperfusion injury (IRI). A recent systematic review and meta-analysis of DCC kidney transplant outcomes was performed that included 51 studies from 1992 to 2020.^3^ For DCC recipients compared to DNC, there was increased risk of primary non-function (relative risk [RR] = 1.43; 95% confidence interval [CI] = 1.26 to 1.62) and delayed graft function (DGF) (RR = 2.02; 95% CI = 1.88 to 2.16). Graft loss was higher at 1 year (RR = 1.13; 95% CI = 1.08 to 1.19) and death-censored graft loss (RR = 1.10; 95% CI = 1.04 to 1.16), as well as 1-year mortality (RR = 1.10; 95% CI = 1.01 to 1.21). The distinction between controlled vs uncontrolled DCC was not consistent across studies. Despite higher risk in the first year after transplant, overall, long-term DCC kidney transplant outcomes were similar to DNC. Accepting even older DCC donor kidneys has been associated with a substantial survival benefit compared to continuing chronic dialysis.^4^

**Figure 1.**
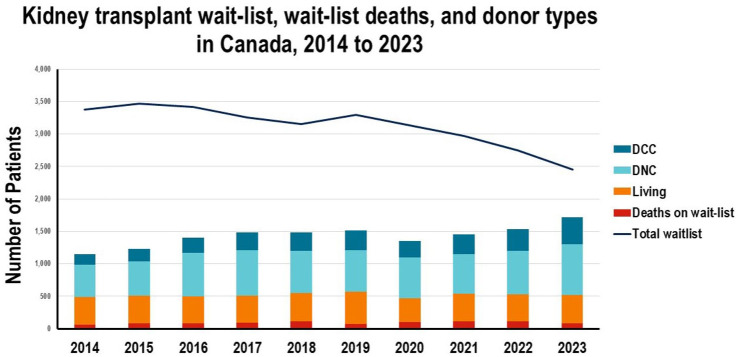
Waitlist for kidney transplant in Canada has been declining in recent years, but the demand for transplants still far exceeds availability.^6^ *Note.* DCC = death determination by circulatory criteria; DNC = death determination was by neurologic criteria.

The DCC activity has already had a tremendous impact on access to transplantation. The use of DCC kidneys in Canada has steadily increased from about 20% a decade ago, to now over one third of all deceased kidneys transplanted (412 of 1198 kidney transplants in 2023).^5^ Nevertheless, 29,906 Canadians were on dialysis in 2023, of whom 2448 people were waiting for a transplant and 85 deaths while waiting for a transplant (Figure 1).^6^ These statistics show that there remains an unmet need.

This narrative review discusses the underlying mechanisms of IRI in the context of DCC, impact of donor characteristics, advances in controlled DCC protocols to reduce the risk of ischemic injury, and opportunities to further expand DCC to meet the demand for kidney transplantation among adults and children.

## Methods

This is a focused review and critical appraisal of existing literature on the mechanisms of kidney IRI, consideration of sex in experimental studies relevant to DCC kidney transplantation, ex vivo perfusion strategies, and pediatric kidney transplant considerations.

### Ischemia-Reperfusion Injury in Donation After Circulatory Death

#### Impact of ischemia-reperfusion on kidney tubular and endothelial cells

Following circulatory death, the kidney undergoes an unavoidable period of warm ischemia characterized by the abrupt cessation of blood flow and a rapid depletion of oxygen and nutrients.^7^ This hypoxic state forces cells to switch to anaerobic metabolism resulting in intracellular acidosis and decreased ATP production.^8^ Subsequently, Na^+^K^+^ ATPase activity is inhibited leading to a rise in intracellular Na+ ions with consequent edema. While the initial ischemic insult affects the entire kidney, the outer medulla, particularly the S3 segment of the proximal tubule and the thick ascending limb, exhibit greater vulnerability due to their high metabolic demands and limited oxygen supply.^9^ At this point, only a small quantity of cells is lost. It is the ensuing reperfusion that paradoxically exacerbates the initial ischemic damage giving rise to IRI.^10^ The reperfusion phase is characterized by a surge in oxidative stress as the sudden reintroduction of oxygen leads to mitochondrial dysfunction and subsequent accumulation of reactive oxygen species, toxic molecules that damage cellular components, including lipids, proteins, and DNA.^10^ Endothelial dysfunction characterized by impaired vascular tone regulation and increased permeability further compromise the microcirculation leading to tissue hypoxia.^10^ Pathways contributing to the loss of functional cells include cell death programs such as apoptosis, necrosis, necroptosis, pyroptosis, and ferroptosis.^11^

#### Immunologic responses to ischemia-reperfusion injury: the innate immune system

Damaged and dying renal cells release damage-associated molecular patterns (DAMPs) that are recognized by pattern recognition receptors, such as toll-like receptors (TLRs) on immune cells. This leads to the production of pro-inflammatory cytokines and chemokines triggering a robust inflammatory response. The innate immune response in kidney IRI includes TLR signaling and complement system activation.^12^ Kidney transplant IRI is associated with increased expression of TLR4 by the injured graft renal tubular cells in rodent models.^13-15^ In humans, TLR4 is upregulated in allografts from deceased donors compared with living donors.^16^ The injured graft expresses and releases endogenous TLR4 ligands such as pro-inflammatory high-mobility group box chromosomal protein 1 (HMGB1).^16,17^ Consequently, activation of TLR4 signaling results in MyD88-dependent activation of nuclear factor kappaB (NF-κB), pro-inflammatory cytokine production including interleukin (IL)-6, IL-1β, and tumor necrosis factor (TNF)-a^18^ and is implicated in leukocyte adhesion and infiltration into injured tissues by promoting the expression of adhesion molecules such as intercellular adhesion molecule-1 (ICAM-1) and E-selectin.^19^ The HMGB1 can also contribute to the pro-inflammatory response by stimulating activated neutrophils to form neutrophil extracellular traps (NETs), whereby neutrophils release their cytotoxic nuclear contents, exacerbating the inflammatory response.^20^ In addition, TLR4 loss-of-function mutations in human allografts were associated with improved immediate graft function.^16^ Furthermore, a rodent model with donor MyD88 deficiency was used to evaluate the relationship between innate immunity and DGF, demonstrating improved graft function, inhibition of neutrophil infiltration, and decreased IL-6 expression.^21^ Thus, innate immunity, and more specifically TLR4 signaling, is implicated as an important determinant of DGF.

Another mediator of IRI in kidney transplantation involves the alternative pathway of complement activation.^12^ Kidney IRI is associated with C3 deposition along the tubular basement membrane, and rodent models with tubular epithelial cell C3aR and C5aR deficiency exhibited attenuated kidney injury.^22^ A comparison of gene expression profiles between allografts from living and deceased donors at implantation (before reperfusion) demonstrated increased expression of complement genes from deceased donors; pre-implantation complement gene expression was also associated with early and late graft function.^23^ Finally, deceased donor kidneys can exhibit a local thrombo-inflammatory response involving complement activation associated with graft dysfunction that is not seen in living donor kidneys.^24^ The complement pathway affects not only the donor organ but the recipient as well.^25^Consequently, the immune response after transplant including TLR signaling and complement activation plays a role in DGF and longer-term graft function.

Beyond the immediate disruption of cellular function, activation of innate immunity and inflammatory cell infiltration also promotes antigen presentation and the subsequent induction of adaptive immune responses. In the context of kidney transplantation, this introduces risk for subsequent immunologic insults including acute rejection and the development of chronic allograft dysfunction which impacts long-term graft survival.^7^ Altogether, the hemodynamic, metabolic, and immunologic disturbances triggered by IRI result in acute tubular necrosis. Prolonged warm ischemia further intensifies cellular damage, increases the release of inflammatory mediators, exacerbates microvascular injury, and impedes the immediate functional recovery of the transplanted kidney manifesting as post-transplant acute kidney injury (AKI)/DGF.^8^

### Sex-Based Differences in Ischemia-Reperfusion Injury and Allograft Outcomes

Biological sex is increasingly being recognized as an important modifier of IRI and AKI.^26,27^ In humans, males with AKI display higher rates of mortality than females.^28,29^ Moreover, a meta-analysis of 83 studies demonstrated that female sex reduces the risk of hospital-associated AKI.^30^ In the context of DCC transplantation, recipients of male donors were at increased risk of DGF by meta-regression.^3^ Recipients of male DCC donors also have longer warm ischemia time (WIT).^31^

Supporting the clinical evidence, studies in porcine and rodent models have consistently shown that male animals have a lower tolerance to IRI than females.^32-34^ The fact that male kidneys display more pronounced lesions has evoked a predominance of male-cantered studies in IRI.^26,35^ This idea is also reflected in 15 experimental studies of DCC (published after 2014). Twelve of these studies were conducted in a porcine model of contralateral nephrectomy and auto-transplantation, as a model of DCC. Of these, 11/12 employed male pigs,^36-46^ 1/12 employed female pigs,^47^ and none of them included both sexes. The predominance of male sex was also evident when DCC outcomes and perfusion methods were investigated in rats^48-50^ (Table 1).

**Table 1.** Evaluation of Sex Inclusion in Preclinical Studies Relevant to Donation After Death Determined by Circulatory Criteria (DCC).

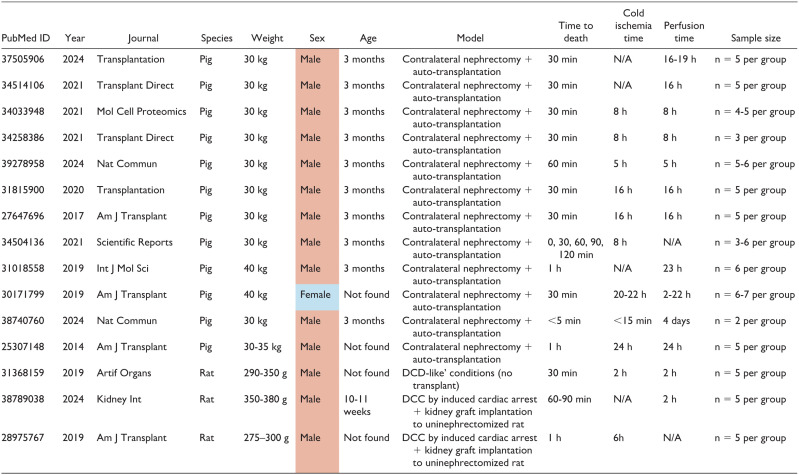

At the molecular level, investigators have studied biological effects linked to the sex of the cell, finding that male sex enhances glucose and glutamine breakdown in proximal tubular epithelial cells (PTECs).^51,52^ This is relevant in transplantation, since altered PTEC metabolism is emerging as one of the overarching processes that drives allograft IRI. Indeed, the metabolic profiling of human kidney biopsies revealed that glycolysis and glutaminolysis in PTECs of DNC and DCC cases are associated with increased severity of post-transplant AKI/DGF and mortality rates.^53,54^ In addition to sex chromosomes, sex-based effects can be ascribed to the action of sex hormones. Improved renal IRI tolerance in female rodents dissipated upon estrogen reduction through ovariectomy, suggesting that estrogens are protective in IRI.^33^ Conversely, gonadectomy attenuated IRI lesions in male rodents, and this effect was reverted by administration of the androgens dihydrotestosterone (DHT) or testosterone, altogether supporting a deleterious role of androgens in IRI.^55,56^ The different effects of sex hormones in IRI could be explained by the different events triggered by each hormone in PTECs. For instance, treatment with DHT but not 17β-estradiol (the main effector estrogen) increased the rates of glycolysis, oxidative stress, and apoptosis in PTECs. Moreover, this effect was more pronounced in male- than in female-derived cells, suggesting that the responsiveness to hormone stimulation is modified by cell sex.^52^ In turn, 17β-estradiol has been associated with improved mitochondrial biogenesis and function, and with upregulation of protective factors, such as the antioxidant protein NRF2.^57,58^ These studies were conducted under normoxia, but whether sex hormones modulate the metabolic maladaptation and subsequent injury of PTECs under hypoxia remains unexplored.

Based on the evidence of sex-based differences in IRI and DCC outcomes, future experimental models of hypoxia and IRI should include kidney cells and animals of both sexes, and tackle sex hormone-related mechanisms, to better evaluate the impact of sex on data outcomes. This could provide evidence to support clinical research investigating sex-specific therapeutic approaches in DCC transplantation.

### Current Policies Around the Use of Donation After Death Determined by Circulatory Criteria Kidneys for Transplantation

Multiple studies have shown an association between the donor (WIT) and DGF. For instance, in a cohort study involving 18 065 recipients of deceased donor kidney transplants in the Eurotransplant cohort study—1059 DCC and 17,006 brain-dead donor (DNC) kidney recipients—every 10-minute increase in WIT was associated with a 20% increase in graft failure (hazard ratio [HR] = 1.20; 95% confidence interval [CI] = 1.03 to 1.42).^59^ Notably, in a multivariable model that included both DCC and DNC kidneys, the association between DCC vs DNC and subsequent graft failure was not statistically significant if WIT was included in the model, suggesting that WIT mediates the increased risk of graft failure associated with DCC vs DNC kidneys. However, other studies have not demonstrated an association between WIT and graft outcomes.^60,61^ The discordance in the literature may be due to differences in the baseline risk for graft function in the donor kidney. For instance, in studies where WIT was not associated with graft failure, it may be that DCC kidneys with the longest WIT were obtained from donors who were younger or healthier, and therefore, the baseline risk for graft failure was low. Consistent with this hypothesis, a cohort study of 28 032 DCC kidney-alone transplants between January 2010 and December 2021 in the United States, investigators examined the association of WIT with death-censored graft failure, with stratification by the Kidney Donor Risk Index (KDRI).^62^ The median WIT in this study was 26 minutes. Of interest, the authors identified that increasing WIT was associated with an increased risk for death-censored graft failure in the subset of kidneys with KDRI >1.14 but not in those with KDRI between 0.78 and 1.14, compared with the reference group of KDRI ≤0.78.

In attempts to limit WIT and consequent IRI and post-transplant AKI/DGF, many organ donation organizations set thresholds for the allowable time to death (TTD) after withdrawal of life-sustaining treatment (WLST) in controlled DCC. While thresholds vary globally,^63-65^ TTD is typically no more than 1 or 2 hours after WLST. If death does not occur within this period, organs will not be procured, and intensive care unit (ICU) will continue to provide end-of-life care. This TTD threshold is based on expert opinion and does not account for the underlying risk of graft failure. Taken together, consideration of the baseline risk of graft failure and careful risk stratification can better inform clinical decision-making regarding utilization of DCC kidneys.

### Opportunities to Expand Kidney Donation After Circulatory Death

**Figure 2.**
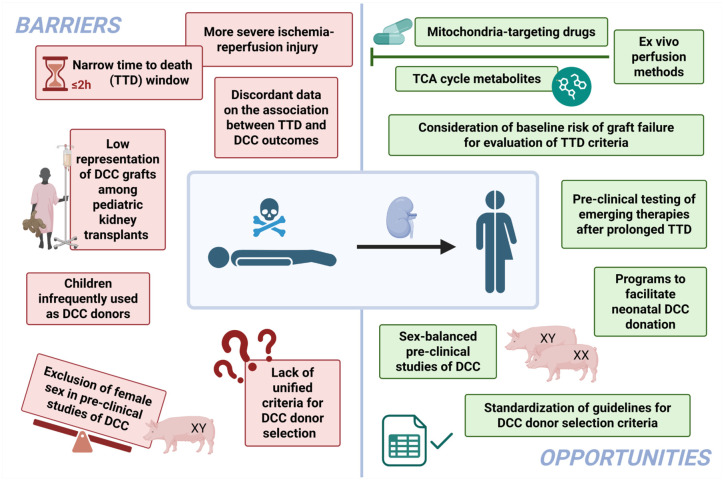
Current barriers and potential opportunities to the continued expansion of controlled donation after circulatory death. *Note.* DCC = donation after death determined by circulatory criteria; TCA = tricarboxylic acid; TDD = time to death.

Appreciating the growing understanding of IRI in the context of DCC transplantation and existing barriers, there may be opportunities to further expand controlled DCC through more detailed evaluation of thresholds for TTD and implementation of ex vivo perfusion strategies. There are also opportunities to expand pediatric access to DCC to address some of this unmet need. These are summarized in Figure 2 and discussed in more detail below.

#### Expanding time to death thresholds for donation after death determined by circulatory criteria

A set TTD does not consider functional warm ischemic time (FWIT), which is the period of organ hypoperfusion and hypoxia from the start of the donor’s physiological decline to organ preservation.^66^ The FWIT is typically estimated based on the onset of donor physiological parameters suggestive of hypoperfusion or hypoxia (eg, systolic blood pressure <50 mm Hg, oxygen saturation <80%) to asystole. Although FWIT may be a better predictor of organ injury and DCC outcomes than total WIT (the period between WLST therapies and organ preservation, including FWIT), definitions of FWIT are inconsistent, and there are limited data on its associations with clinical outcomes.^67,68^ Establishing standardized criteria for the onset of FWIT would increase consistency between studies and facilitate incorporation of this measure in DCC decision-making.

One study found that among DCC kidneys that were procured, the percentage of donors with FWIT >30 minutes did not increase with increasing TTD up to 4 hours from a traditional threshold of 2 hours.^69^ This suggests that it may be reasonable to have longer thresholds for TTD as long as FWIT remains low. Indeed, in the United Kingdom, the national standard for donation after circulatory death (DCD) minimum wait time was set to a minimum of 3 hours.^64^ Analysis of DCC under a 3-hour minimum wait policy included an estimated 5635 transplants were performed from donors with TTD <30 minutes, 663 from donors with TTD of 30 to 59 minutes, 582 from donors with TTD of 60 to 119 minutes, 261 from donors with TTD of 120 to 179 minutes, and 42 from donors with TTD of over 180 minutes. Donor TTD was not associated with recipient 12-month estimated glomerular filtration rate (eGFR) (adjusted coefficient = −0.25, 95% CI = −0.68 to 0.19, *P* = .27).^70^ The increased threshold was also associated with a 14.1% increase in the number of DCD kidney transplants.

#### Ex vivo preservation methods to improve allograft outcomes

Ex vivo machine perfusion emerged in the early 2000s as a novel preservation technology for assessing and storing marginal grafts. It was first utilized successfully in lungs procured from a DCC donor in 2001 in Lund, Sweden,^71^ and its wider implementation quickly facilitated hundreds of lung transplants that would not have otherwise happened.^72,73^ The use of ex vivo machine perfusion has been gradually extended to other solid organs including hearts,^74^ livers,^75^ and kidneys.^36^ The technology is revolutionizing the field of allogeneic organ transplantation and has the potential of facilitating the utilization of more DCC kidneys.

The high metabolic demands of the kidney limit its functional preservation and explain why temperature and oxygen are critical parameters when considering optimal methods for preservation. At normothermia (37°C), kidneys are metabolically active, but ex vivo preservation is limited to hours.^76^ During hypothermia (4-8°C), kidneys can be preserved for up to 24 hours but are metabolically inactive and suffer hypothermic injury.^77^ Recent research has predominantly focused on 3 perfusion methods: non-oxygenated hypothermic machine perfusion (non-oxHMP), oxygenated hypothermic machine perfusion (oxHMP), and normothermic ex vivo kidney perfusion (NEVKP). During non-oxHMP, a cold (4-8°C) acellular preservation solution circulates through the renal artery by a perfusion pump. To perform oxygenation of the organs during perfusion (oxHMP), a membrane oxygenator is integrated into the perfusion circuit.^78^ The NEVKP relies on the circulation of a warmed (37°C), oxygenated, red cell-based perfusate through the kidney to maintain near-physiological conditions. All 3 methods (either alone or sequentially combined) have shown to improve the function and survival of porcine^37,45,46^ and human kidney grafts,^79-81^ when compared to the classic method of static cold storage (SCS).

Mechanistically, the benefits of non-oxHMP relative to SCS were associated with an increase in endothelial nitric oxide synthase (eNOS) activating phosphorylation by adenosine monophosphate-activated protein kinase (AMPK), together with an increase in NO-dependent vasodilation of renal arteries at the end of preservation.^37^ In turn, molecular profiling at the gene, protein, and metabolite level supports that NEVKP can attenuate critical ischemia-driven metabolic alterations in the graft, namely the increase in renal glycolysis and lactate excretion, as well as the decrease in renal tricarboxylic acid (TCA) cycle metabolism, mitochondrial respiration, and levels of adenosine triphosphate (ATP) and mitochondrial proteins.^43,44^ These effects were further enhanced by AP39, a compound that targets and stimulates mitochondria by donating a hydrogen sulfide group.^42^ Given these observations, novel strategies have emerged with the purpose of maintaining TCA cycle activity (and in consequence, mitochondrial respiration) during ex vivo kidney perfusion. To this end, de Haan and colleagues recently implemented the use of a cell-free perfusate supplemented with TCA cycle intermediates to DNC and DCC kidney grafts.^36^ This approach allowed a 4-day ex vivo metabolic preservation of both porcine and human kidney grafts, thus prolonging previously reported perfusion times.

The accumulating and compelling evidence supporting the benefits of ex vivo kidney preservation is accelerating its clinical implementation. Kang et al^82^ published an updated systematic review and meta-analysis of machine perfusion in human DCC kidneys, which included 19 randomized clinical trials from November 2018 to July 2023. The meta-analysis concluded that non-oxHMP reduces DGF risks and suggested that NEVKP shows promise in DCC donation. The same concepts were reviewed recently by Sonnenberg et al,^83^ who highlighted the need for more studies examining oxHMP and NEVKP in human kidneys. The authors also pointed to remaining gaps in implementation associated with increased organ non-utilization, such as poor interpretation of pump parameters and the complex logistics needed for utilizing ex vivo perfusion devices in a routinary fashion.^84,85^ Of note, some important limitations at the level of preclinical research were not addressed in these reviews. All preclinical studies testing ex vivo perfusion methods in DCC kidneys identified in the review study were conducted after short periods of warm ischemia (30 min to 2 h) (Table 1), perhaps with the rationale that longer warm ischemia times lead to a more severe degree of renal dysfunction that may be irreversible even with subsequent perfusion.^39^ However, this possibility has not been reevaluated in the context of new perfusate compositions showing extended benefits, such as the ones containing AP39^42^ or TCA metabolites.^36^ Supporting this idea, ex vivo perfusion in lungs significantly lowered graft injury after 3 hours of warm ischemia.^86^ Another relevant gap is the sex bias in the preclinical testing of ex vivo perfusion methods, possibly explained by the male-predominant nature of IRI studies. Hypothermic and normothermic methods have been repeatedly studied in male animals, with only 1 team reporting the use of oxHMP and non-oxHMP in females (Table 1). To our knowledge, NEVKP still remains to be tested in females, and there is still a paucity of studies including animals of both sexes. Addressing these limitations may help expand the DCC donor pool while revealing sex-specific insights on the applicability and molecular mechanisms associated with different kidney preservation strategies.

### Donation After Circulatory Death in the Pediatric Context

In Canada, increasing rates of DCC transplantation have predominantly occurred among adult recipients. The DCC allografts account for <5% of all pediatric kidney transplants in the United States, compared to 24% of adult kidney transplants.^87^ Currently, many pediatric transplant programs across Canada and globally do not accept allografts from DCC donors, due to concerns about poor long-term allograft survival.^88^ Although children are prioritized by organ allocation systems in most jurisdictions, there is evidence of a growing mismatch between the rising number of waitlisted pediatric transplant candidates and the static donor pool. In the United States, the number of children waitlisted for organ transplant has increased by 32% from 2011 to 2022 and >20% of children now wait for more than 3 years to undergo transplantation.^87^ This problem is exacerbated by declining rates of living donation to pediatric recipients in many countries.^87,88^ As a result, 1% to 2% of children die per year on the waitlist before they can reach transplantation.^87^

In pediatric transplantation, maximizing allograft survival is a clear priority, to delay the need for future kidney replacement therapy, repeat transplantation, and reduce sensitization events. However, prolonged time on the waitlist increases a child’s risk of death and morbidity.^89^ Prolonged childhood dialysis duration is associated with substantial adverse medical, neurocognitive, and growth outcomes.^90^ Utilization of DCC allografts in pediatric recipients has been shown to reduce the amount of time that a child spends on the waitlist.^87,91^ Furthermore, it is estimated that expanded use of DCC allografts could double the number of pediatric transplants performed each year.^91^ Children without options for living donation, who are highly sensitized, or experience high disease- or treatment-related morbidity may particularly benefit from earlier transplantation with DCC allografts.

The use of DCC allografts for pediatric transplantation is supported by several recent studies which demonstrate similarly high rates of long-term allograft and patient survival among pediatric recipients of DCC vs DNC kidney and liver transplants.^91-95^ Among 285 children that received DCC kidney transplants and 1132 propensity score-matched DNC controls, there was no difference in 10-year allograft (59% vs 52%) or patient survival (96% vs 96%).^93^ Critically, this study found that DCC transplantation was associated with a significant survival benefit, compared to remaining on the waitlist (adjusted HR = 0.44, 95% CI = 0.21 to 0.92). These findings are consistent with other reports, which have found no difference in allograft and patient survival among children receiving DCC vs DNC kidney transplants.^91,92^ While long-term outcomes of DCC kidney transplantation in children are reassuring, rates of DGF are reported to be up to 25% among DCC recipients, compared to ~10% among DNC recipients.^91-93^,^96^ Still, DGF in pediatric DCC recipients is substantially less common than among adults and has not been shown to adversely impact long-term allograft function.^92^

Children are also infrequently used as DCC donors. In Canada and the United States, DCC accounts for 8% of all pediatric deceased donors, compared to 21% to 32% of all deceased donors.^87,97,98^ Yet, several studies have found that long-term allograft outcomes are similar among recipients of pediatric vs adult DCC kidney and liver transplants.^99-101^ Furthermore, it is estimated that expanded access to pediatric DCC donation would increase the overall kidney donor pool by more than 20%, shortening wait times for both DCC and DNC transplantation.^97,102^ In a single-center implementation study of a pediatric DCC program, 17 of 53 (32%) children evaluated for potential DCC met eligibility criteria, and 7 (41%) families consented for DCC donation.^103^ Implementation of this pediatric DCC program increased organ donation rates by 58%. Another study found that 9% of children that died in the pediatric intensive care unit met criteria for DCC donation, which could have increased rates of kidney and liver transplantation by more than half.^102^ It is estimated that 8% of neonatal intensive care unit deaths would meet DCC criteria.^104^ Establishing programs to facilitate neonatal DCC donation could also increase the donor pool, since brain death is less common in neonates.^105^

Although neonatal and pediatric DCC donation is considered medically and ethically viable, widespread global variation exists in access to and utilization of pediatric DCC programs.^98^ In Canada, 73% of pediatric intensive care units have policies in place to routinely support pediatric DCC donation, but there are substantial differences between centers in practices for identifying, referring, and approaching potential DCC donors.^106^ There is also a lack of consensus among Canadian organ donation organizations on criteria for pediatric DCC, which results in the exclusion of many potential donors.^107^ Canadian clinical practice guidelines for controlled pediatric DCC support expanded access to standardized pediatric DCC services across Canada.^107^

## Limitations

This narrative review only included articles written in English. Study quality was not formally assessed. Discussion points were influenced by the author’s areas of expertise.

## Conclusion

This review has identified various barriers at the clinical and preclinical levels that limit the utilization of DCC donor selection while providing research- and policy-based insights on how these barriers could be addressed (Figure 2).

At the clinical level, the utilization of DCC grafts is challenged by the narrow TTD window of eligibility of DCC grafts (≤2 h), highly driven by the more severe IRI associated with DCC grafts, and by discordant data on the association between TTD and DCC outcomes. These clinical barriers are further accentuated by the lack of unified criteria for DCC donor selection across health systems nationally and internationally. The FWIT may be a stronger, more specific predictor of adverse DCC outcomes than total WIT. Establishing standardized criteria defining FWIT would support the use of this metric in future clinical research and DCC donor evaluation. Low utilization of DCC organs is particularly common among pediatric recipients, despite evidence of increasing wait times and comparable outcomes of DCC vs DNC organs in this population. Although consensus guidelines have been established for pediatric DCC donation in Canada,^98^ several ethical, social, and logistical barriers (eg, informed consent, public perception, lack of standardized protocols, selection criteria, and infrastructure) limit its utilization. Collective initiatives are warranted to mitigate these challenges, such as the consideration of baseline risk of graft failure for evaluation of TTD criteria, the development of programs to facilitate neonatal and pediatric DCC donation, and the harmonization of guidelines for DCC donor selection criteria for children and adults.

At the preclinical level, the systematic exclusion of the female sex and lack of sex-disaggregated data in experimental studies of DCC have limited the clinical translatability of the research findings. There is a need for greater inclusion of females in animal and clinical research, with appropriate exploration of sex-based differences. This is supported by regulatory guidance in Canada and the United States promoting inclusive research designs. Moreover, the investigation of IRI mechanisms and treatment responses has been mostly conducted based on the current ≤2 hours TTD threshold. The coupling of normothermic ex vivo graft perfusion with the infusion of TCA cycle metabolites or mitochondria-targeting agents is emerging as a promising therapeutic approach to prevent IRI in DCC. This review encourages the future testing of these therapeutic strategies upon prolonged TTD windows and highlights the urgent need for sex-balanced preclinical studies of DCC in kidney transplantation research.
